# Recommendations of the Moroccan Society of Rheumatology (SMR) for Diagnostic Management of Spondyloarthritis (SpA) and Psoriatic Arthritis (PsA)

**DOI:** 10.31138/mjr.230929rtm

**Published:** 2023-07-31

**Authors:** Ibtissam Bentaleb, Lamia Oulkadi, Nada Jaouad, Abdellah El Maghraoui, Redouane Niamane, Imane El Bouchti, Saloua Larhrissi, Linda Ichchou, Saadia Janani, Fatima Abourazzak, Nessrine Akasbi, Mariam Erraoui, Samia Karkouri, Ahmed Bezza, Ihsane Hmamouchi, Rachid Bahiri

**Affiliations:** 1 Department of Rheumatology A, El Ayachi Hospital, Ibn Sina University Hospital, Salé, Morocco,; 2 Private Medical Office, Rabat, Morocco,; 3 Department of Rheumatology, Military Hospital Avicenne, Mohammed VI University Hospital, Marrakech, Morocco,; 4 Department of Rheumatology, Arrazi University Hospital, Marrakech, Morocco,; 5 Private Medical Office, Rabat, Morocco,; 6 Department of Rheumatology, Mohammed VI University Hospital, Oujda, Morocco,; 7 Department of Rheumatology, Ibn Rochd University Hospital, Casablanca, Morocco,; 8 Department of Rheumatology, Tanger-Tetouan-Al Hoceima University Hospital, Tanger, Morocco,; 9 Department of Rheumatology, Hassan II University Hospital, Fès, Morocco,; 10 Department of Rheumatology, University Hospital Hassan II-Souss Massa, Agadir, Morocco,; 11 CARBONE research team, LARISS Laboratory, FMPA, Ibn Zohr University, Agadir-Morocco,; 12 Department of physical medicine and rehabilitation, El Ayachi Hospital, Ibn Sina University Hospital, Salé, Morocco,; 13 Department of Rheumatology, Military Hospital Mohammed V, Ibn Sina University Hospital, Rabat, Morocco,; 14 Health Sciences College, International University of Rabat (UIR), Rabat, Morocco,; 15 Department of Rheumatology, Provincial Hospital of Temara, Temara, Morocco

**Keywords:** recommendations, spondyloarthritis, psoriatic arthritis, Morocco

## Abstract

In 2017, the Moroccan Society of Rheumatology (SMR) issued guidelines for the treatment of severe ankylosing spondylitis. The emergence of new therapeutic classes, the update of international guidelines for axial SpA and psoriatic arthritis, and the diagnostic and therapeutic challenges encountered by rheumatologists has led to the development of recent SMR guidelines for the management of SpA patients. A group-work included rheumatologists, specialists in physical medicine and rehabilitation, and epidemiologists from various sectors was tasked with writing these recommendations based on a literature review, then adapting them to the national context. Thus, 33 recommendations were selected and organized into two sections: diagnostic and therapeutic. The diagnostic section included three general principles and fourteen recommendations. The first, second, and third recommendations concerned the definition and diagnostic criteria for psoriatic arthritis and psoriatic arthritis. In the management of SpA, Recommendation 4 prioritized the importance of opportunity windows. The recommendation5 concerned the diagnostic and prognostic significance of HLAB27. The sixth and seventh recommendations related to imaging of the sacroiliac joints and the spine. The eighth recommendation focuses on the diagnosis and evaluation of perivascular thrombosis activity. The ninth and tenth recommendations concerned the evaluation of SpA activity and psoriatic arthritis. The eleventh and twelfth recommendations concern the evaluation of function and structural progression. The recommendation number thirteen concerned the diagnosis and treatment of coxitis. Recommendation 14 focuses on the most common co-morbidities and extra-rhinitological manifestations observed in SpA.

## INTRODUCTION

Spondyloarthritis (SpA) is a complex disease with different phenotypic presentations.^1^ It is the cause of severe disability and excess mortality with a significant socio-professional and economic impact. The patient’s quality of life and prognosis will be enhanced by early diagnosis and collaborative management.

The recommendations of the Moroccan Society of Rheumatology (SMR) are based on the recent recommendations of ACR2019,^2^ SFR 2018,^3^ The latest ASAS-EULAR recommendations (2022) and a rigorous synthesis of the literature.^4^

The aim of this work is to provide specialists with a practical tool to improve the management of patients with SpA and to make this management in Morocco agreeable, compliant, and accessible to all rheumatologists.

## METHODS

### Steering committee

The convener (RB) invited a steering committee to develop new SMR recommendations for the management of patients with SpA. Three fellows performed the SLR. The convenor, the expert rheumatologist and methodologist supervised the fellows’ SLR work. The steering committee included eleven rheumatologists, a specialist in physical medicine and rehabilitation (SK), an epidemiologist (IH) and one patient representative (BZ). The SLRs focused on the recent databases regarding the classification criteria of SpA and PsA and their evaluation; the imaging of spine and sacro-iliac joint; the importance of window of opportunity in the management of SpA; the update of diagnosis and management of the main comorbidities and extra-rheumatologic manifestations during the SpA. Further discussion will focus on the SLR data and suggestions from the steering committee, in order to develop the updated recommendations and voting.

### Delphi process

Two weeks before the first voting round, there was a mail-briefing session where the TF (Taskforce) learned about the decision rules and the level of evidence. The level of agreement of TF members was reported during each round of voting, using a numerical rating scale of one (totally disagree) to nine (totally agree). Participants provided also qualitative comments. The two Delphi rounds were performed through live online meetings moderated by the methodologist (IH). All the members of TF have discussed results during each round. The OMERACT recommendations served as a guide to move from one Delphi round to another and for selecting the final statement. 5 The consensus was accepted if >70% of the members voted in favour of the recommendation at the first round, second round was accepted if the score > 70% high agreement (7–9) and < 15% low agreement.^1–3^

## RESULTS

### Principle 1

*-Even if its clinical presentation varies greatly depending on the patient, spondyloarthritis is a potentially serious and disabling chronic disease that can deteriorate the quality of life and reduce life expectancy.*
*-It can cause functional restrictions and limitations with a socio-professional impact and an economic repercussion either directly through the cost of the disease or indirectly through the loss of productivity.*

Numerous studies have shown the physical, mental and socioeconomic burden caused by the alteration of the functional capacity of patients.^6–7^ SpA is responsible for diagnostic delay, absenteeism and sick leave in young patients. Early diagnosis with disease control, prevention of functional impairment and work adjustment are the solutions to reduce the socio-economic impact of the disease.^7^

### Principle 2

*Spondyloarthritis is a condition that requires a multidisciplinary approach involving medical, paramedical staff and others.*

*The rheumatologist is the specialist who coordinates the management of spondyloarthritis.*

*This management must be global, allowing the control of the various dimensions of the disease.*

SpA is a heterogeneous disease with rheumatological and extra-articular manifestations requiring multidimensional management, by the attending rheumatologist and other specialists (gastroenterologists, dermatologist, cardiologist, etc.).^3,8–9^

The management of spondyloarthritis must be a global approach. Medication, physical therapy, education, and surgery are some of the components that should be included in the management of spondyloarthritis. These components should be enhanced by social and professional interventions. In fact, adopting a patient-centred approach and making sure that the effects of SpA are taken into account across the physical, mental, occupational, and social spectrum, instead of just focusing on disease activity, should improve care and help patients to achieve real-life improvements.

### Principle 3

*The initial therapeutic objective is to obtain and maintain clinical remission or a low disease activity in order to improve the patient’s quality of life, prevent structural damage and ensure optimal autonomy and social reintegration.*

*To achieve this goal, a Treat to Target (T2T) approach can be implemented and adapted to each patient by including the different clinico-biological parameters of the disease, the extra-articular manifestations and comorbidities as well as the NSAIDs intake.*

The “T2T” approach is widely accepted in rheumatoid arthritis with better functional and structural results.^10^ However, in SpA we do not have formal evidence demonstrating the effectiveness of this concept. Even though the TICOSPA study didn’t find a statistically significant difference between T2T and standard care for the primary judgment “ASAS,” it did come to the conclusion that it’s important to use a standardised measurement tool that shows how active the disease is and to set a goal for this measurement tool to have a more rigorous approach.^8,11^

In the same case, Smollen et al. proposed recommendations for the “T2T” strategy based mainly on these 3 principles^8^:

- Adopt the T2T strategy as early as possible in the course of the disease.
- Aim for an acceptable status (remission or low disease activity) in terms of disease activity based on a valid composite index.
- Modify and/or intensify treatment if improvement has been observed after 12 weeks and/or if the goal has not been achieved after 6 months of treatment.

This therapeutic objective must be adapted to the clinical presentation of the disease, but also take into account the patient’s comorbidities, expectations, life plans or fears. This objective represents the basis of the shared therapeutic project.^12^

### Principle 4: Definition and terminology

*-Spondyloarthritis is a heterogeneous chronic inflammatory rheumatic disease characterised by the predominant involvement of the entheses, with axial and/or peripheral rheumatological manifestations as well as extra-articular manifestations associated with a particular genetic background.*
*-Several phenotypes can be distinguished:*
  - *Radiographic axial form*
  - *Non-radiographic axial form*
  - *Peripheral form (articular and enthesitic)*
*-The phenotype can be better characterised by adding the possible concomitant extra-articular manifestations.*

This principle has integrated the new terminology that has been proposed, in order to better describe the clinical phenotype of SpA.^9^

### Recommendation 1: Diagnosis

*The diagnosis of the different phenotypic forms of spondyloarthritis is based on the opinion of the expert rheumatologist, supported by a combination of anamnestic, clinical, biological and imaging evidence.*

*The diagnosis of non-radiographic axial spondyloarthritis is based on the opinion of the rheumatologist and the presence of HLAB27 or MRI sacroiliitis.*

*The rheumatologist may use classification criteria systems. The most commonly used are the ASAS criteria.*

The 2009 ASAS criteria for axial SpA classify patients with a compatible clinical picture as having nrAxSpA with sacroiliitis on MRI or even without imaging confirmation for HLA B27-positive patients.^13^ The management of spondyloarthritis must be a global approach. Indeed, since their validation, the ASAS criteria which include MRI data of the sacroiliac joints (SIJ), have been shown in large cohorts to have good sensitivity and specificity compared to the AMOR and ESSG criteria, even with the addition of the MRI criterion of SIJ.^13–14^

### Recommendation 2: Diagnosis of psoriatic arthritis

*- Psoriatic arthritis is a polymorphic chronic rheumatic disease that may be included as a member of the SpA spectrum. Its clinical presentations are varied.*
*- The diagnosis of the different forms of psoriatic arthritis is based on the opinion of the expert rheumatologist, supported by a combination of anamnestic, clinical, biological and imaging evidence.*
*- The CASPAR criteria and the ASAS criteria for peripheral SpA may be used.*
*- Skin psoriasis should be systematically searched for in any context of rheumatic inflammatory disease.*

Classification criterion systems, such as the ASAS criteria for peripheral SpA and CASPAR, can assist the rheumatologist establish the diagnosis of PsA in addition to his or her experience and the collected data.

One study has shown that CASPAR criteria have comparable sensitivity and specificity to the ASAS criteria for peripheral SpA and other criteria specific to PsA.^15–16^

### Recommendation 3: Window of opportunity

*Early management of SpA, optimally within the window of opportunity, regardless of SpA phenotype, should be targeted for rapid disease control. The level of evidence and the duration of this window are not yet clear.*

Several studies have shown the existence of a window of opportunity in SpA, they are all unanimous in the favourable prognosis of the disease due to the early introduction of a specific drug treatment.^17–18^ One of the objectives of the window of opportunity in non-radiographic SpA could be to slow down the development of structural damage, particularly ossification phenomenon.^19^ Recent MRI studies support this concept by showing that under anti-TNFa treatment, pure inflammatory lesions disappear without giving rise to a syndesmophyte, whereas more complex lesions, associating fatty elements with inflammation, are associated with this ossification.^19^

The determination of the duration of this window remains the subject of much debate. However, there is no formal demonstration that can determine the period when therapeutic management has the maximum chance of success; Furthermore, studies have shown the therapeutic effectiveness of NSAIDs and anti-TNFa in recent forms of SpA.^20^

### Recommendation 4: HLAB 27

*In case of an uncertain clinical situation, looking for HLAB 27 is of a diagnostic value, but its absence does not eliminate the diagnosis. HLA B27 is also of prognostic value; its presence is associated with an early disease onset, increased inflammation of the sacroiliac joints on MRI, and the occurrence of uveitis. It is also a predictive factor of the response to anti-TNF in axial forms and in uveitis.*

The DESIR cohort showed that HLAB27 was correlated with an early onset of the disease with shorter diagnostic time, higher frequency of radiographic sacroiliitis, increased inflammation of the SIJ on MRI.^21^ A national multicentre study concluded that the presence of HLA B27 is strongly associated with early disease onset, higher activity, and a high prevalence of coxitis and uveitis.^22^ The post hoc analysis of the randomised GO-RAISE and ACERT trial concluded that the HLAB27+ profile is associated with a better response to antiTNFa.^23^

In Morocco, since previous national studies focused mainly on the prevalence of HLAB27 in small number of patients with AS, its exact prevalence in patients with SpA (including axial and peripheral form) remains unknown. In recent national multicentre study HLAB27 was found in 44.5% of SpA patients.^22^ These findings are consistent with the prevalence observed in the MENA region.^24–26^ In the same study, the prevalence of HLAB27 in axial form was 47.21%.^22^ These findings correspond to the prevalence of axSpA found in published studies in the Arab and Middle Eastern region (42.7%84%). ^24–26^

### Recommendation 5: Diagnosis of Sacroiliitis

*The diagnosis of radiographic sacroiliitis follows the modified New York criteria and is defined by the presence of bilateral grade 2 or unilateral grade 3 sacroiliitis.*

*In the absence of sacroiliitis on standard radiography or in doubtful cases, MRI is recommended. It allows an early diagnosis.*

*CT may be recommended too for differential diagnosis in some cases.*

The diagnosis of axial SpA is based on sacroiliac involvement on standard radiography. This is recommended as the first line imaging test for the diagnosis of SpA.^27^ In some situations, young age of onset, short duration of disease progression, or in case of questionable sacroiliitis on standard radiography, SI MRI is recommended as a first alternative.^27^ In fact, many studies have shown the contribution of SI MRI in the early diagnosis of SpA.^27–28^

CT could be recommended as a third-line in the absence of sacroiliitis on standard radiography and when MRI of SIJ is inconclusive or cannot be performed. 27 CT is more recommended in the differential diagnosis to distinguish between inflammatory and degenerative phenomena.^27^

Virtual non-calcium dual-energy CT is a new scanning technique to study oedema.^29^ Low-dose CT is a promising tool in SpA, but its place in the early diagnosis of the disease remains to be elucidated.

### Recommendation 6: Spinal Imaging

*- A standard spinal X-ray is recommended for any patient with inflammatory spinal pain. It could also be recommended for the follow-up of the structural evolution.*
*- MRI of the spine is not routinely recommended for the positive diagnosis of SpA. It can be ordered in certain specific clinical situations. It has not yet been shown to be useful in the follow-up of the disease.*

The experts considered it important to indicate a radiograph of the lumbar spine in cases of inflammatory low back pain, given the additional arguments that it could bring to the diagnosis in the presence of syndesmophytes. MRI of the SIJ combined with MRI of the spine is useful in the diagnosis of SpA, especially when there is a strong clinical suspicion and when MRI of the SIJ is inconclusive.^30^ MRI of the spine would seem to be more useful for differential diagnosis, evaluation of degenerative spine, anatomical variants or certain pathologies that may mimic spinal inflammation during spondyloarthritis such as metastatic or septic spine.^31^

### Recommendation 7: Diagnosis and activity of peripheral enthesitis

*- Enthesitis can be seen in all phenotypes. It is part of the ASAS criteria. Its detection is essentially clinical.*
*- Specific scores can be used to evaluate its activity. The most commonly used are the MASES, LEI and SPARCC scores. They are more used in clinical research.*
*- The sites most affected are those of the lower limbs, particularly the calcaneal, which is responsible for talalgia and patellar pain.*
*- In certain situations, ultrasound can be used as a confirmatory examination*.

The diagnosis of enthesitis is both an essential element for the positive diagnosis and a parameter for the evaluation of the disease activity.^32^ Several indices have been proposed for the evaluation of enthesitis activity, the MASES, LEI and SPARCC scores. MASES remains the most used index because, in addition to its good metrological properties, it takes into account the major peripheral and axial entheseal sites affected by SpA.^32^ Ultrasound coupled with Doppler remains the examination of choice to confirm both the diagnosis and follow the evolution of enthesitis.^32^

### Recommendation 8: Assessment of SpA activity

*-Composite assessment scores adapted to each form should be used to assess disease activity:*
*-The preferred scores for predominantly axial forms are the ASDAS or BASDAI.*
*-For predominantly peripheral forms, the scores derived from the DAS are used.*
*-The recommended frequency of follow-up will be determined by the treating rheumatologist according to the disease activity and the clinical context.*

Given the complexity and heterogeneity of the clinical aspects of SpA, the evaluation of its activity through a simple and valid “gold standard” means proves difficult. Thus, the evaluation of SpA is multidimensional using scores associating the main components of the disease. The ASDAS activity score remains more reliable than the BASDAI. Indeed, studies have shown that the ASDAS score correlates strongly with different biomarkers of inflammation and changes in signs on MRI compared to BASDAI.^8^

### Recommendation 9: Assessment of psoriatic arthritis activity

*- The assessment of activity in psoriatic arthritis is often complex. It must be adapted to the clinical form.*
*- A dermatological score (PASI) coupled with a rheumatological score (ASDAS, DAS ...) must be used in the presence of cutaneous psoriasis.*
*- Other scores for evaluating the activity of psoriatic arthritis can be used, such as the Disease activity index for psoriatic arthritis (DAPSA) or the Minimal Disease Activity (MDA), but their use in current practice remains limited.*

The assessment of activity in psoriatic arthritis (PsA) remains subject to much discussion given the heterogeneity of the disease. Several composite scores have been proposed for the evaluation of PsA: CPDAI, GRACE, MDA, PASDAS, DAPSA, ASDAS and DAS 28.^8^

The choice of the composite score will depend on the clinical presentation; so far there is no consensus on the choice of the composite score to measure the activity of PsA. According to expert opinion, composite scores that integrate different aspects of the disease summarised in a single numerical value provide a more accurate and comprehensive estimate of disease activity than individual variables related to each condition.^8,15^

### Recommendation 10: Assessment of function

*- Assessment of the functional impact of SpA to patients should be performed using validated indices (BASFI, HAQ) according to the phenotype.*
*- The evaluation of hip function, in case of coxitis, must be regular and specific using an adapted score (Lequesne).*
*- Collaboration with physical medicine and rehabilitation specialists is recommended to optimize management. It is useful at all stages of the disease.*

Several studies have proven the validity of scores for assessing the quality of life of patients with SpA. Through large observational and cohort studies, the ASAS-HI score has demonstrated its reliability and good reactivity as well as its correlation with the other scores, in particular ASQoL, BASFI and ASDAS.^33^ The HAQ has also proven its good validity and reliability in SpA with a good correlation with the SF-36.^34^

### Recommendation 11: Assessment of structural progression

*- Assessment of structural progression in axial and peripheral SpA should be performed by regular radiographs. It is based on the opinion of the expert rheumatologist.*
*- Several radiographic scores, BASRI, SASSS, mSASSS, RASSS, have been validated in axSpA. Their use is not mandatory in current practice.*
*- The frequency of this evaluation should be adapted to each clinical situation.*

There are several radiographic scores for assessing structural progression in SpA (BASRI, SASSS, mSASSS, RASSS).^30^ The mSASSS score is the most widely used because of its validity, reliability, and feasibility.^35^ Alternatively, radiographic progression in the spine could be correlated to ASDAS-CRP score. A German cohort showed an association between the ASDAS-CRP and the mSASSS score.^36^

### Recommendation 12: Coxitis

*- Coxitis is frequent and specific in our context, requiring early diagnosis. It is indicative of a severe form of the disease, given its functional impact.*
*-Diagnosis and follow-up are based on clinical examination and standard radiography.*
*-Ultrasound allows the detection of joint effusion and synovitis. It also allows for local procedures to be performed. In the early stages, MRI can be useful.*
*- The response to NSAIDs and biological treatment is often insufficient, hence the interest in local procedures. In the terminal stage, prosthetic hip replacement is the only alternative.*

Involvement of the coxofemoral joint may reveal the course of SpA. Its frequency remains high in the Moroccan SpA patients (25–53%).^38^ The correlation between the occurrence of coxitis, severe axial involvement and the presence of HLA B27 has been demonstrated in several studies.^38^

### Recommendation 13: Comorbidities and extra-articular manifestations

*- Early and regular management of comorbidities and extra-articular manifestations is essential to optimize disease control and prognosis.*
*- Particular attention should be paid to certain conditions in collaboration with the organ specialist, and careful screening for comorbidities and extra-articular manifestations is recommended.*

Extra-articular manifestations of SpA and comorbidities are frequent and sometimes severe. They must be detected at the time of diagnosis and during follow-up, in collaboration with the organ specialist in charge.

### R13a: Ophthalmologic disease

*- Acute anterior uveitis is the most frequent extra-articular manifestation in SpA and can be the first symptom leading to the diagnosis.*
*- Because of its severity, it must be systematically detected by questioning. Its management requires close collaboration between rheumatologists and ophthalmologists.*

Uveitis is the most frequent extra-articular involvement in AS. It may be seen in 20–30% of cases during the evolution of the disease.^39^ In Morocco, data on the prevalence of uveitis in SpA are not clearly established; a recent multicentre study found a prevalence of 23.7%.^22^ In 90% of cases, uveitis is anterior, acute and unilateral.^39^

### R13b: Cardiovascular disease

*- Cardiovascular risk is increased in patients with SpA. Certain disease-related factors increase this risk, including the inflammatory process and the use of NSAIDs.*
*- Cardiac damage related to SpA is dominated by valvulopathy and conduction disorders.*
*- Screening must be systematic, and rapid management is mandatory in coordination with a cardiologist.*

Several studies have shown that CVD and risk factors such as hypertension, metabolic syndrome, and diabetes mellitus are more prevalent in patients with SpA.^40^ The ASAS-COMOSPA study showed high figures of CVD especially in Northern European and American countries (10%) with a cardiovascular risk unlike in Morocco where the prevalence of CVD was 1%.^41^

### R13c: Bone disease

*- Osteoporosis is a frequent problem in SpA.*
*-An osteoporotic fracture risk assessment with regular densitometric monitoring is recommended.*
*- The interpretation of BMD must take into account possible confounding factors (ankylosed spine or presence of coxitis).*

The risk of osteoporosis and vertebral fractures is well established through several studies.^42^ The prevalence of these fractures varies between 10 and 17%. 42 In the COMOSPA study, osteoporosis was the most frequent comorbidity in patients with SpA with a prevalence of 13.4%.^41^

### R13d: Gastrointestinal disease

*- In addition to the classic association of SpA with chronic inflammatory bowel disease (IBD), asymptomatic bowel lesions may exist.*
*- Management requires collaboration between rheumatologists and gastroenterologists and must take into consideration the effect of certain rheumatological treatments, particularly NSAIDs, on enteropathy.*
*- The indication for certain csDMARDs or certain biologics must take into account, the overall context and be made in consultation between the rheumatologist and the gastroenterologist.*

Apart from the known association between SpA and IBD,^43^ noted in 5 to 10% of patients, endoscopic studies have shown that asymptomatic intestinal lesions were present in 30 to 50% of SpA patients.^44^ In addition, several studies have shown that gastrointestinal inflammatory disease is associated with rheumatologic disease activity, inflammation of the SIJ on MRI, and increased biomarkers of GI inflammation.^43–45^

### R13e: Lung disease

*- Pulmonary disease in SpA is most often asymptomatic. It is dominated by restrictive disorders, apical pulmonary fibrosis and interstitial damage.*
*- The frequency and type of pulmonary investigation should be decided according to the context and in collaboration with the pulmonologist.*
*- Infectious complications must be regularly screened and smoking cessation is strongly recommended.*

In a Moroccan Series, in asymptomatic patients, apical pulmonary fibrosis was identified in 6.9% of cases, emphysema in 18.1%, bronchiectasis in 10.8%.^46^ The SpA patient with pulmonary damage should benefit from close monitoring to watch for possible complications.^46–47^

### R13f: Renal disease

*- Renal disease in SpA is uncommon but prognostic. It is essentially dominated by amyloidosis, often late in the course of the disease and associated with a severe form of the disease, IgA nephropathy and lithiasis.*
*- There are factors that aggravate renal function, particularly long-term use of NSAIDs.*
*- It is necessary to carry out an appropriate renal assessment at the time of diagnosis and on a regular basis during the follow-up of the disease.*

The renal manifestations encountered in SpA are essentially represented by amyloidosis, IgA nephropathy, lithiasis and analgesic nephropathy. Renal involvement is uncommon but prognostic, with prevalence ranging from 4.3 to 35%.^47^

A standard renal assessment at the time of diagnosis and during the follow-up of SpA will allow early management of renal damage and consequently an improvement of the prognosis of the disease.^47–48^

## DISCUSSION

The SpA is a heterogeneous disease requiring multidimensional management.

Concerning the “T2T approach”, admittedly, we do not have formal evidence demonstrating the effectiveness of this concept in SpA, but several studies are unanimous on the interest of setting a target on SpA measurement tools to have a more rigorous approach.^8,10–12^ The task force of the latest ASAS-EULAR recommendations for the management of axial spondyloarthritis emphasized that a treatment target should be used as a guideline, but should only result in intensifying immunosuppressive treatment if both the physician and the patient are convinced of the residual inflammatory activity’s presence and other (contextual) factors do not prevent such an intensification.^4^ They include fibromyalgia and other comorbidities, which might possibly affect the assessment of disease activity.^4^

The assessment of activity in PsA is still subject to much debate due to the heterogeneity of the disease. The EULAR 2019 recommendations call for assessment of the therapeutic response of PsA by using the DAPSA and MDA scores.^15^ Regarding the monitoring of patient with axSpA, the ASDAS score seems to be the best suitable tool for assessing disease activity, and now is recommended for monitoring patients with axSpA.^4^

Furthermore, among the new recommendations of ASAS-EULAR, ASDAS was selected as the instrument used to determine a patient’s eligibility for treatment with b/tsDMARDs (ASDAS 2.1) and concerning the continuation of therapy (improvement 1.1).^4^ In fact, for more than a decade of comprehensive experience with ASDAS and the collected data demonstrating its superiority, its selection was a must. When the ASDAS cannot be used, the BASDAI is preferable to no instrument at all. Nonetheless, we recommend that every attempt is made to implement the ASDAS in routine clinical practice.

Given the importance of imaging in the diagnosis and follow-up of patients with SpA, the experts developed recommendations that addressed the diagnosis of sacroiliitis, focusing on the different alternatives in the absence of SI damage on standard radiography and the value of spinal imaging in the diagnosis and follow-up in recommendation 5 and 6 respectively. In The ASAS-EULAR recommendations 2022, and due to its high costs, the frequent use of the MRI is not recommended for monitoring since its efficacy for this purpose is still unclear.^4^

The latest ASAS EULAR recommendations stated that in order to detect the structural damage, the radiographs of the spine should be performed every 2 years, since the progression of structural damage occurs at a slow rate.^4^ HLAB27 has been the subject of numerous studies which are all unanimous on the strong correlation between this antigen, early disease onset, higher activity and increased inflammation of SIJ on MRI.^21–22^

Coxitis is a marker of disease severity. Its prevalence remains high in Morocco (29%),^37^ which is why the expert group devoted a coxitis review to the importance of early detection and follow-up of this condition.

The frequency of extra-articular manifestations and the severity of comorbidities have attracted particular attention. The main objective is to ensure a rigorous and regular early detection and management of these conditions in collaboration with the organ specialist.^49^ The followings are two examples of the published articles on the consensus statements and recommendations on psoriatic arthritis and SpA from Arabic countries:

In March 2018, a group of 14 rheumatologists, including members of the Kuwaiti Association of Rheumatology (KAR), have developed adapted guidelines to local patients regarding the management of SpA.^50^ The 41 recommendations are organized into five categories highlighting essential definitions and treatment concepts for both axial and peripheral spondyloarthritis. These recommendations are not intended to be full treatment guidelines, and should only be used appropriately to assist in clinical decision-making.^50^

Furthermore, an expert group of key opinion leaders from the UAE has recently developed consensus statements for the evaluation and treatment of PsA. The present consensus statements are in accordance with established international norms for the many components of PsA, emphasising in particular the evaluation of PsA and nonpharmaceutical therapy for PsA. These consensus statements can help healthcare practitioners in the UAE evaluate and treat patients with PsA more effectively.^51^

## CONCLUSION

The ultimate goal of this work is to provide the rheumatologist with an adapted and easy-to-use tool in order to improve the management of patients with SpA. Updates will be considered at a later date according to therapeutic advances and the evolution of knowledge and the results of ongoing studies.

**Table 1.** Overarching principles of the SMR recommendations for the diagnostic management of Spondyloarthritis including Psoriatic arthritis with level of agreement.

**Overarching Principles**	**Level of agreement**
*-Even if its clinical presentation varies greatly depending on the patient, spondyloarthritis is a potentially serious and disabling chronic disease that can deteriorate the quality of life and reduce life expectancy.* *-It can cause functional restrictions and limitations with a socio-professional impact and an economic repercussion either directly through the cost of the disease or indirectly through the loss of productivity.*	**8,4± 1.4**
*-Spondyloarthritis is a condition that requires a multidisciplinary approach involving medical, paramedical staff and others.* *-The rheumatologist is the specialist who coordinates the management of spondyloarthritis.* *-This management must be global, allowing the control of the various dimensions of the disease.*	**9 ± 0**
*-The initial therapeutic objective is to obtain and maintain clinical remission or a low disease activity in order to improve the patient’s quality of life, prevent structural damage and ensure optimal autonomy and social reintegration.* *-To achieve this goal, a Treat-to-Target (T2T) approach can be implemented and adapted to each patient by including the different clinico-biological parameters of the disease, the extra-articular manifestations and comorbidities as well as the NSAIDs intake.*	**8,7± 0,8**
*-Spondyloarthritis is a heterogeneous chronic inflammatory rheumatic disease characterised by the predominant involvement of the entheses, with axial and/or peripheral rheumatological manifestations as well as extra-articular manifestations associated with a particular genetic background.* *-Several phenotypes can be distinguished:* *□ Radiographic axial form* *□ Non-radiographic axial form* *□ Peripheral form (articular and enthesitic)* *-The phenotype can be better characterised by adding the possible concomitant extra-rheumatological manifestations.*	**8,9 ± 0.3**

**Table 2.** The SMR recommendations for the diagnostic management of Spondyloarthritis including Psoriatic arthritis with level of agreement.

**Recommendations**	**Level of agreement**
*R1: Diagnosis* *-The diagnosis of the different phenotypic forms of spondyloarthritis is based on the opinion of the expert rheumatologist, supported by a combination of anamnestic, clinical, biological and imaging evidence.* *-The diagnosis of non-radiographic axial spondyloarthritis is based on the opinion of the rheumatologist and the presence of HLAB27 or MRI sacroiliitis.* *-The rheumatologist may use classification criteria systems. The most commonly used are the ASAS criteria.*	**8,8 ± 0,4**
*R2: Diagnosis of psoriatic arthritis* *- Psoriatic arthritis is a polymorphic chronic rheumatic disease that may be included as a member of the SpA spectrum. Its clinical presentations are varied.* *- The diagnosis of the different forms of psoriatic arthritis is based on the opinion of the expert rheumatologist, supported by a combination of anamnestic, clinical, biological and imaging evidence.* *- The CASPAR criteria and the ASAS criteria for peripheral SpA may be used.* *- Skin psoriasis should be systematically searched for in any context of rheumatic inflammatory disease.*	**8,4 ±1**
*R3: Window of opportunity* *Early management of SpA, optimally within the window of opportunity, regardless of SpA phenotype, should be targeted for rapid disease control. The level of evidence and the duration of this window are not yet clear.*	**8,6± 0,6**
*R4: HLAB 27* *In case of an uncertain clinical situation, looking for HLAB 27 is of a diagnostic value, but its absence does not eliminate the diagnosis. HLA B27 is also of prognostic value; its presence is associated with an early disease onset, increased inflammation of the sacroiliac joints on MRI, and the occurrence of uveitis. It is also a predictive factor of the response to anti-TNF in axial forms and in uveitis.*	**8,3± 1,4**
*R5: Diagnosis of Sacroiliitis* *The diagnosis of radiographic sacroiliitis follows the modified New York criteria and is defined by the presence of bilateral grade 2 or unilateral grade3 sacroiliitis.* *In the absence of sacroiliitis on standard radiography or in doubtful cases, MRI is recommended. It allows an early diagnosis.* *CT may be recommended too for differential diagnosis in some cases.*	**8,5± 1,2**
*R6: Spinal Imaging* *- A standard spinal X-ray is recommended for any patient with inflammatory spinal pain. It could also be recommended for the follow-up of the structural evolution.* *- MRI of the spine is not routinely recommended for the positive diagnosis of SpA. It can be ordered in certain specific clinical situations. It has not yet been shown to be useful in the follow-up of the disease.*	**8,6± 0,6**
*R7: Diagnosis and activity of peripheral enthesitis* *- Enthesitis can be seen in all phenotypes. It is part of the ASAS criteria. Its detection is essentially clinical.* *- Specific scores can be used to evaluate its activity. The most commonly used are the MASES, LEI and SPARCC scores. They are more used in clinical research.* *- The sites most affected are those of the lower limbs, particularly the calcaneal, which is responsible for talalgia and patellar pain.* *- In certain situations, ultrasound can be used as a confirmatory examination.*	**8,4±1,2**
*R8: Assessment of SpA activity* *-Composite assessment scores adapted to each form should be used to assess disease activity:* *-The preferred scores for predominantly axial forms are the ASDAS or BASDAI.* *-For predominantly peripheral forms, the scores derived from the DAS are used.* *-The recommended frequency of follow-up will be determined by the treating rheumatologist according to the disease activity and the clinical context.*	**8,8±0,4**
*R9: Assessment of psoriatic arthritis activity* *- The assessment of activity in psoriatic arthritis is often complex. It must be adapted to the clinical form.* *- A dermatological score (PASI) coupled with a rheumatological score (ASDAS, DAS ...) must be used in the presence of cutaneous psoriasis.* *- Other scores for evaluating the activity of psoriatic arthritis can be used, such as the Disease activity index for psoriatic arthritis (DAPSA) or the Minimal Disease Activity (MDA), but their use in current practice remains limited.*	**8,4 ± 0,8**
*R10: Assessment of function* *- Assessment of the functional impact of SpA to patients should be performed using validated indices (BASFI, HAQ) according to the phenotype.* *- The evaluation of hip function, in case of coxitis, must be regular and specific using an adapted score (Lequesne).* *- Collaboration with physical medicine and rehabilitation specialists is recommended to optimize management. It is useful at all stages of the disease.*	**7,8±2,2**
*R11: Assessment of structural progression* *- Assessment of structural progression in axial and peripheral SpA should be performed by regular radiographs. It is based on the opinion of the expert rheumatologist.* *- Several radiographic scores, BASRI, SASSS, mSASSS, RASSS, have been validated in axSpA. Their use is not mandatory in current practice.* *- The frequency of this evaluation should be adapted to each clinical situation.*	**8,5±0,8**
*R12: Coxitis* *- Coxitis is frequent and specific in our context, requiring early diagnosis. It is indicative of a severe form of the disease, given its functional impact.* *- Diagnosis and follow-up are based on clinical examination and standard radiography.* *-Ultrasound allows the detection of joint effusion and synovitis. It also allows for local procedures to be performed. In the early stages, MRI can be useful.* *- The response to NSAIDs and biological treatment is often insufficient, hence the interest in local procedures. In the terminal stage, prosthetic replacement is the only alternative*	**8,3±1**
*R13: Comorbidities and extra-articular manifestations* *- Early and regular management of comorbidities and extra-articular manifestations is essential to optimize disease control and prognosis.* *- Particular attention should be paid to certain conditions in collaboration with the organ specialist, and careful screening for comorbidities and extra-articular manifestations is recommended.*	**8,5 ±1,2**
*R13a: Ophthalmologic disease* *- Acute anterior uveitis is the most frequent extra-articular manifestation in SpA and can be the first symptom leading to the diagnosis.* *- Because of its severity, it must be systematically detected by questioning. Its management requires close collaboration between rheumatologists and ophthalmologists.*	**8,7± 0,9**
*R13b: Cardiovascular disease* *- Cardiovascular risk is increased in patients with SpA. Certain disease-related factors increase this risk, including the inflammatory process and the use of NSAIDs.* *- Cardiac damage related to SpA is dominated by valvulopathy and conduction disorders.* *- Screening must be systematic, and rapid management is mandatory in coordination with cardiologist.*	**8,1±1,5**
*R13c: Bone disease* *- Osteoporosis is a frequent problem in SpA.* *-An osteoporotic fracture risk assessment with regular densitometric monitoring is recommended.* *- The interpretation of BMD must take into account possible confounding factors (ankylosed spine or presence of coxitis).*	**9 ±0,3**
*R13d: Gastrointestinal disease* *- In addition to the classic association of SpA with chronic inflammatory bowel disease (IBD), asymptomatic bowel lesions may exist.* *- Management requires collaboration between rheumatologists and gastroenterologists and must take into consideration the effect of certain rheumatological treatments, particularly NSAIDs, on enteropathy.* *- The indication for certain csCSDMARDs or certain biologics must take into account, the overall context and be made in consultation between the rheumatologist and the gastroenterologist.*	**8,6±1,2**
*R13e: Lung disease* *- Pulmonary disease in SpA is most often asymptomatic. It is dominated by restrictive disorders, apical pulmonary fibrosis and interstitial damage.* *- The frequency and type of pulmonary investigation should be decided according to the context and in collaboration with the pulmonologist.* *- Infectious complications must be regularly screened and smoking cessation is strongly recommended.*	**8,7 ± 0,6**
*R13f: Renal disease* *- Renal disease in SpA is uncommon but prognostic. It is essentially dominated by amyloidosis, often late in the course of the disease and associated with a severe form of the disease, IgA nephropathy and lithiasis.* *- There are factors that aggravate renal function, particularly long-term use of NSAIDs.* *- It is necessary to carry out an appropriate renal assessment at the time of diagnosis and on a regular basis during the follow-up of the disease.*	**8,7 ± 0,6**

## ABBREVIATIONS

ACR: American College of Rheumatology
ASAS: Assessment of SpondyloArthritis International Society
ASAS-HI: Assessment of SpondyloArthritis International Society- health Index
ASDAS: Ankylosing Spondylitis Disease Activity Score
ASQoL: Ankylosing Spondylitis Quality of Life Questionnaire
Amor and ESSG: The Amor and European Spondyloarthropathy Study Group
Anti-TNF-a: Anti-tumour Necrosis Factor alpha
BASDAI: Bath Ankylosing Spondylitis Disease Activity Index
BASFI: Bath Ankylosing Spondylitis Functional Index
BASRI: Bath Ankylosing Spondylitis Radiology Index
BMD: Bone Mineral Density
CASPAR: Classification for Psoriatic Arthritis
CPDAI: Composite Psoriatic Disease Activity Index
CSDMARDs: Conventional Synthetic Disease-Modifying Anti-Rheumatic Drugs
CT: Computed Tomography
CVD: Cardiovascular disease
DAPSA: Disease Activity index for PSoriatic Arthritis
DAS: Disease Activity Score
GRACE: GRAppa Composite scorE
HAQ: Health Assessment Questionnaire
IBD: Inflammatory Bowel Disease
LEI: Leeds Enthesitis Index
MASES: Maastricht Ankylosing Spondylitis Enthesitis Score
MDA: Minimal Disease Activity
MENA: Middle East and North Africa
mSASSS: Modified Stoke Ankylosing Spondylitis Spine Score
MRI: Magnetic Resonance Imaging
NSAIDs: Non-steroidal anti-inflammatory drugs
PASDAS: Psoriatic ArthritiS Disease Activity Score
PASI: Psoriasis Area Severity Index
PsA: Psoriatic arthritis
RASSS: Radiographic Ankylosing Spondylitis Spinal Score
SASSS: Stoke Ankylosing Spondylitis Spine Score
SFR: French Society of Rheumatology
SIJ: Sacroiliac joints
SLR: Systematic Literature Review
SMR: Moroccan Society of Rheumatology
SPARCC: Spondyloarthritis Research Consortium of Canada
SpA: Spondyloarthritis
